# The Role of Macrophage-Inducible C-Type Lectin in Different Stages of Chronic Liver Disease

**DOI:** 10.3389/fimmu.2020.01352

**Published:** 2020-07-07

**Authors:** Robert Schierwagen, Frank E. Uschner, Cristina Ortiz, Sandra Torres, Max J. Brol, Olaf Tyc, Wenyi Gu, Christian Grimm, Stefan Zeuzem, Andreas Plamper, Philipp Pfeifer, Sebastian Zimmer, Christoph Welsch, Liliana Schaefer, Karl P. Rheinwalt, Joan Clària, Vicente Arroyo, Jonel Trebicka, Sabine Klein

**Affiliations:** ^1^Department of Internal Medicine I, University Hospital, Goethe University, Frankfurt, Germany; ^2^Department of Internal Medicine I, University of Bonn, Bonn, Germany; ^3^Department for Bariatric, Metabolic and Plastic Surgery, St. Franziskus-Hospital, Cologne, Germany; ^4^Department of Medicine II, Heart Center, University Hospital Bonn, Bonn, Germany; ^5^Centre for Pharmacy Frankfurt/ZAFES, Institute for Pharmacology and Toxicology, University Hospital, Goethe University, Frankfurt, Germany; ^6^European Foundation for the Study of Chronic Liver Failure, Barcelona, Spain; ^7^Department of Medical Gastroenterology and Hepatology, Odense University Hospital, Odense, Denmark; ^8^Department of Mechanical Biology, Institute for Bioengineering of Catalonia, Barcelona, Spain

**Keywords:** ACLF, bacterial translocation, fibrosis, inflammation, NASH

## Abstract

The macrophage-inducible C-type lectin (mincle) is part of the innate immune system and acts as a pattern recognition receptor for pathogen-associated molecular patterns (PAMPS) and damage-associated molecular patterns (DAMPs). Ligand binding induces mincle activation which consequently interacts with the signaling adapter Fc receptor, SYK, and NF-kappa-B. There is also evidence that mincle expressed on macrophages promotes intestinal barrier integrity. However, little is known about the role of mincle in hepatic fibrosis, especially in more advanced disease stages. Mincle expression was measured in human liver samples from cirrhotic patients and donors collected at liver transplantation and in patients undergoing bariatric surgery. Human results were confirmed in rodent models of cirrhosis and acute-on-chronic liver failure (ACLF). In these models, the role of mincle was investigated in liver samples as well as in peripheral blood monocytes (PBMC), tissues from the kidney, spleen, small intestine, and heart. Additionally, mincle activation was stimulated in experimental non-alcoholic steatohepatitis (NASH) by treatment with mincle agonist trehalose-6,6-dibehenate (TDB). In human NASH, mincle is upregulated with increased collagen production. In ApoE deficient mice fed high-fat western diet (NASH model), mincle activation significantly increases hepatic collagen production. In human cirrhosis, mincle expression is also significantly upregulated. Furthermore, mincle expression is associated with the stage of chronic liver disease. This could be confirmed in rat models of cirrhosis and ACLF. ACLF was induced by LPS injection in cirrhotic rats. While mincle expression and downstream signaling via FC receptor gamma, SYK, and NF-kappa-B are upregulated in the liver, they are downregulated in PBMCs of these rats. Although mincle expressed on macrophages might be beneficial for intestinal barrier integrity, it seems to contribute to inflammation and fibrosis once the intestinal barrier becomes leaky in advanced stages of chronic liver disease.

## Introduction

The macrophage-inducible Ca^2+^-dependent lectin receptor (mincle) is a primary component of the innate immune response and acts as a sensor for pathogen-associated molecular patterns (PAMPS) and damage-associated molecular patterns (DAMPS) and is expressed mainly on cell types of the myeloid lineage (e.g., macrophages) (1). Trehalose-6,6-dimycolate (TDM) stands out among the PAMPS that interact with mincle (2). The mycobacterial cell wall glycolipid has strong immunomodulatory functions. Binding of the ligand to mincle leads to interaction with the signaling adapter FC receptor gamma chain (FCER1G) (3). The formed receptor complex enables intracellular signaling molecules to dock on the immunoreceptor tyrosine-based activation motif (ITAM) and thereby transduce signaling in immune cells. The downstream signaling of the complex proceeds inter alia via spleen associated tyrosine kinase (SYK) and the nuclear factor kappa-light-chain-enhancer (NF-κB) to induce the gene expression of pro-inflammatory cytokines, chemokines, and enzymes (4, 5). On the cellular level, mincle stimulation promotes the inflammatory phenotype of mainly M1 macrophages (6, 7).

Mincle is known for its pro-inflammatory properties, especially in non-alcoholic (NASH) and alcoholic steatohepatitis (ASH) (8, 9), and might be involved in the activation of myofibroblasts (8). In NASH, mincle is predominantly reported to be involved in the formation of crown-like structures. In these structures, dying cells are surrounded by infiltrating macrophages, which promote adipose tissue inflammation and fibrosis (10, 11). Similar structures have been described in NASH livers, but not in other etiologies of chronic liver disease (12). To date, only one study has investigated the role of mincle in ASH in a murine model with mild fibrosis. This study identified Kupffer cells, the macrophages of the liver, as the main source for mincle expression. The inhibition of downstream signaling by SYK inhibitors led to decreased production of inflammatory markers (9).

In more advanced stages of chronic liver disease, an impaired intestinal barrier followed by bacterial translocation into the blood circulation are important events in the development from stable liver cirrhosis toward acute-on-chronic liver failure (ACLF) (13). Infiltrating bacteria or bacterial products lead to chronic systemic inflammation (14). Mincle possibly plays an important role in this process in two respects, namely integrity of the intestinal barrier and systemic inflammation. It recognizes gut bacteria that penetrate the Peyer's patches and induces the activation of immune cells to reinforce the intestinal barrier and thus prevents systemic inflammation. Furthermore, mincle deficiency leads to increased bacterial translocation and hepatic inflammation (15). Since ACLF is accompanied by a high short-term mortality of 30% within 28 days (16), targeting mincle in advanced chronic liver disease potentially restores intestinal barrier integrity and reduces bacterial translocation, thereby possibly preventing ACLF development.

Since to date, there are only a few reports published about the role of mincle in fibrosis and none about its role in more advanced stages of chronic liver injury, this study aims to assess the role of mincle in cirrhosis and ACLF in the liver and in other organs that are highly affected by systemic inflammation.

## Materials and Methods

### Human Liver Samples

Liver samples from cirrhotic patients were taken during liver transplantation at the University of Bonn between 1999 and 2005. Liver samples of non-cirrhotic donors were used as controls. This study was approved by the local ethics committee of the University of Bonn (029/13).

Liver samples of NASH patients were taken during bariatric surgery at the St. Franziskus-Hospital (Cologne, Germany) between 2018 and 2019. NASH was diagnosed in liver biopsies independently by two experienced pathologists using the non-alcoholic fatty liver disease activity score after Kleiner (17). Non-alcoholic fatty liver diseases were ruled out for control individuals with <5% of parenchymal steatosis. NASH patients had a Kleiner score of 6 or 7, with at least 33% of parenchymal steatosis, hepatocyte ballooning, lobular inflammation, and grade 2 or 3 fibrosis. This study was approved by the ethics committees of the North Rhine-Westphalian Chamber of Medicine (2017110) and of the University of Bonn (194/17).

Liver samples were immediately stored at −80°C and cut on dry ice to avoid thawing. The studies were performed in accordance with the declaration of Helsinki and patients gave their written informed consent.

### Animals

Twelve-week-old apolipoprotein E knockout mice (ApoE^−/−^, C57BL/6J background; Charles River, Wilmington, USA) were used for induction of experimental NASH.

For induction of chronic liver disease and ACLF, male wild type Sprague Dawley rats with an initial body weight of 180–200 g were used. Rats were fed standard rat chow (Ssniff, Soest, Germany).

All animals received water and chow *ad libitum*. The animals were kept in individually ventilated cages at 22°C with a 12 h day/night cycle. All experiments were performed in accordance with the German Animal Protection Law and the guidelines of the animal care unit at the University of Bonn (Haus für experimentelle Therapie, Bonn, Germany) and approved by the relevant North Rhine-Westphalian state agency for Nature, Environment, and Consumer Protection (LANUV, Germany) (LANUV84-02.04.2014.A137).

### Induction of Experimental NASH and Administration of Mincle Agonist TDB

Mice were fed a high-fat, cholesterol-rich diet (western diet; WD) containing 21% fat (with coconut oil), 19.5% casein, and 1.25% cholesterol (Ssniff, Soest, Germany) for seven weeks to induce NASH as described previously (18–21).

Trehalose-6,6-dibehenate (TDB) is a synthetic analog of the mycobacterial cell wall glycolipid of trehalose-6,6-dimycolate (TDM). TDB (dose 50 μg, Invitrogen, Karlsbad, CA, USA) was injected s.c. once per week for 7 weeks. Control mice received solvent (sodium chloride).

### Induction of Experimental Cirrhosis and Acute-on-Chronic Liver Failure

To induce cirrhosis, bile duct ligation (BDL) was performed as a model for cholestatic liver disease as described previously (22, 23). To induce ACLF, rats received a single intraperitoneal dose of lipopolysaccharide (LPS from *E. coli* O111:B4, Sigma–Aldrich, St. Louis, USA, 6.25 mg/kg body weight) 25 days after BDL and were sacrificed 72 h after injection. BDL and sham-operated rats without LPS injection served as controls. Sodium chloride was used as solvent.

### Tissue and Blood Collection

At the end of the experiment, animals were anesthetized and laparotomy was performed for tissue collection. Liver, kidney, spleen, small intestine, and heart were stored at −80°C until further use as described previously (18, 24). Blood samples were collected in EDTA tubes (Sarstedt, Nümbrecht, Germany) for isolation of peripheral blood mononuclear cells (PBMC). PBMCs were isolated by density gradient centrifugation using Pancoll (PAN-Biotec, Aidenbach, Germany) as described previously (25). Cells were suspended in RPMI 1640 media with 10% fetal calf serum and 10% dimethyl sulfoxide (Gibco, Carlsbad, USA) and stored at −80°C until further use.

Human liver samples were snap-frozen and stored at −80°C following excision as described previously (26).

### Hepatic Hydroxyproline Content

Hydroxyproline content measurement was performed as described previously (24, 26). Briefly, analog segments of 200 mg snap-frozen liver samples were dissolved and homogenized in 12 N hydrochloric acid at 110°C. Homogenized samples were later dissolved in methanol, oxidized with chloramine T, and finally reacted with Ehrlich's reagent. Samples were measured photometrically at 558 nm.

### Quantitative Polymerase Chain Reaction

RNA isolation and quantitative polymerase chain reaction (qPCR) were performed as described previously (24). Briefly, total RNA was isolated with ReliaPrep RNA Miniprep Systems and cDNA synthesis was performed by the ImProm-II Reverse Transcription System (both Promega, Madison, WI, USA). DNase digestion was performed to dispose of genomic DNA. TaqMan gene expression assays (Thermo Fisher Scientific, Waltham, MA, USA) were used (Supplemental Table 1) for qPCR according to the manufacturer's protocol on a 7300 Real-Time PCR System (Applied Biosystems, Foster City, CA, USA). Experiments were carried out in duplicates. Gene expression was calculated by the 2^−ΔΔ*Ct*^ method and results were standardized against 18S rRNA expression. Gene expression levels were shown as x-fold expression compared with the respective control group.

### Transcriptome Analysis

Transcriptome analysis was performed by OakLabs (Hennigsdorf, Germany) using the Agilent Microarray XS (Agilent Technologies, Santa Clara, USA). Briefly, Low Input QuickAmp Labeling Kit (Agilent Technologies, Santa Clara, USA) was used to create fluorescent complementary RNA (cRNA) followed by hybridization to microarrays using the Gene Expression Hybridization Kit (Agilent Technologies, Santa Clara, USA). Fluorescence signals were detected using SureScan Microarray Scanner (Agilent Technologies, Santa Clara, USA).

### Western Blotting

Protein levels were analyzed by Western blot as described previously (24, 26). Briefly, snap-frozen livers were homogenized and diluted. The protein content of homogenates was determined with the DC assay kit (Bio-Rad, Munich, Germany). Forty micrograms of protein samples was subjected to SDS-PAGE under reducing conditions (10% gels), and proteins were blotted on nitrocellulose membranes. The membranes were blocked and incubated with primary antibody against mincle (NBP1-49311, Novus Biologicals, Littleton, USA). Glyceraldehyde-3-phosphate dehydrogenase (GAPDH) served as an endogenous control (sc-166545 for human samples and sc-47724 for rodent samples; both Santa Cruz Biotechnology, Santa Cruz, CA). Membranes were incubated with the corresponding secondary antibody, and blots were developed using enhanced chemiluminescence. Protein quantification was performed by ImageJ (version 1.51q, NIH, USA) and results were corrected for GAPDH levels.

### Statistics

Statistical analyses were performed using Prism V.5.0 (GraphPad, San Diego, CA). Data were expressed as mean ± standard error of the mean (SEM). Groups were tested by Shapiro–Wilk-test for normal distribution. Comparisons between two groups were done by unpaired *t*-test or by non-parametric Mann–Whitney *U t*-tests. Correlations were performed using SPSS V25 (IBM SPSS Statistics for Windows, Version 25.0, Armonk, NY, USA). Statistical analysis of patient characteristics and gene expressions were done by calculation of Spearman's correlation coefficient with *p*-value. *P* < 0.05 were considered statistically significant.

For transcriptome analysis, statistical parameters were computed between groups, and results are shown as log2-fold change and visualized by heatmaps. *P*-values were calculated using paired *t*-test and corrected according to the adaptive Benjamini–Hochberg procedure. A FDR-adjusted *p*-value below 0.05 was considered statistically significant.

## Results

### Mincle in NASH

**Figure 1:**
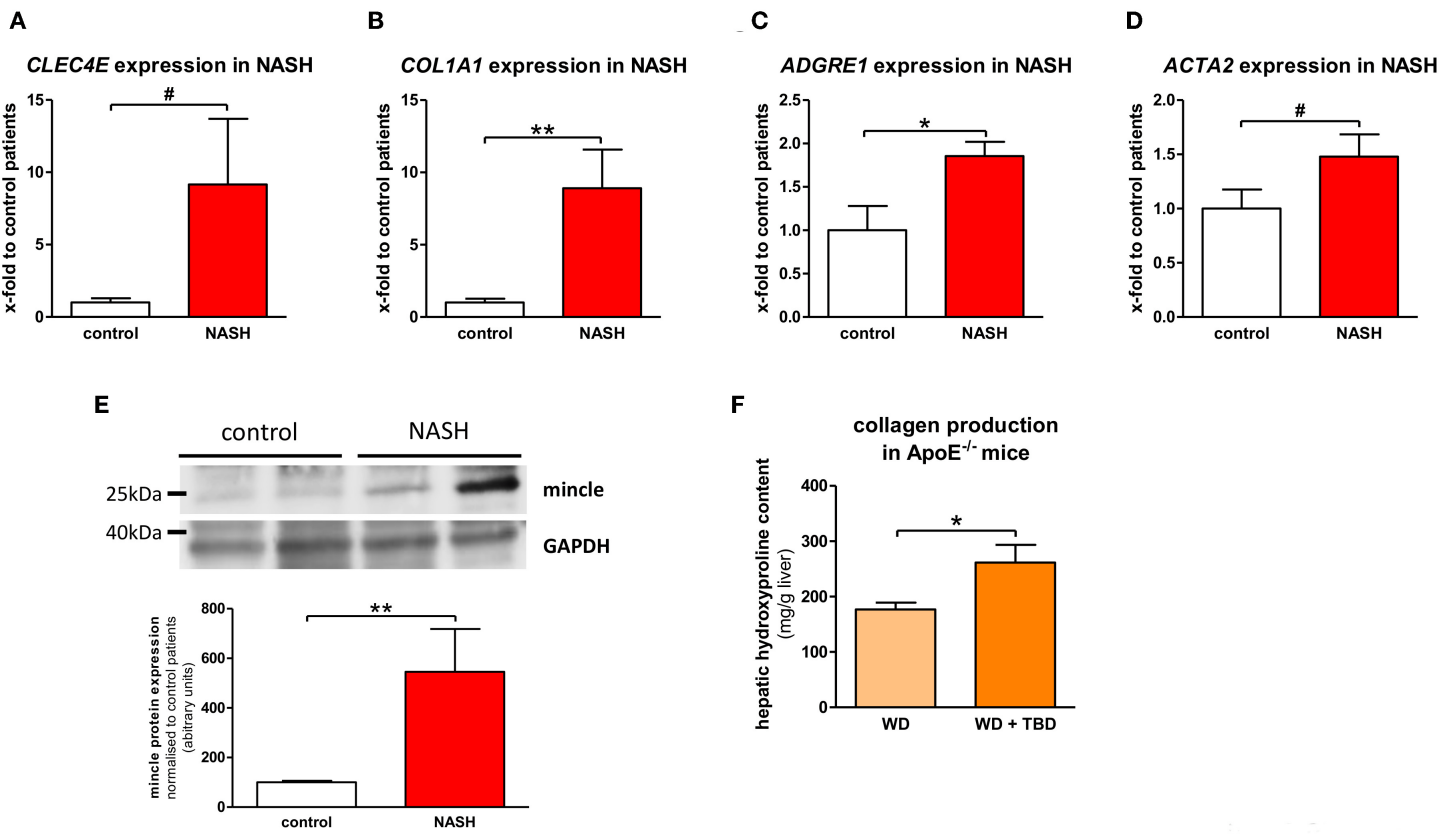
Mincle in NASH. **(A–D)** Gene expression of mincle [gene *CLEC4E*; **(A)**], collagen [gene *COL1A1*; **(B)**], EMR1 [gene *ADGRE1*; **(C)**], and αSMA [gene *ACTA2*; **(D)**] in liver samples of non-alcoholic steatohepatitis (NASH) patients and non-NASH controls (control). **(E)** Representative western blot showing protein levels of mincle in NASH patients and non-NASH controls and quantification of the Western blots. **(F)** Hepatic collagen production, shown by measurement of hepatic hydroxyproline content, in ApoE^−/−^ fed WD with or without TDB treatment. ^*^p < 0.05; ^**^p < 0.01; ^#^*p* < 0.1 using unpaired *t*-test **(A,D)** or non-parametric Mann–Whitney *U t*-test **(B,C,E,F)** after testing for normal distribution.

The expression of mincle (*gene CLEC4E*) was assessed in human liver samples from patients with NASH who underwent bariatric surgery and in liver samples from donors for liver transplantation. Mincle shows a tendency toward upregulation in patients with NASH without reaching statistical significance (Figure 1A). NASH patients show significantly higher fibrogenesis (Figure 1B), as demonstrated by collagen 1 (gene *COL1A1*), and inflammation (Figure 1C) than control patients, as demonstrated by macrophage and Kupffer cell marker EMR1 (gene *ADGRE1*). Furthermore, there is a trend toward upregulation of αSMA (gene *ACTA2*), a surrogate marker of hepatic stellate cell activation (Figure 1D). Interestingly, mincle expression is upregulated with increased collagen 1 expression (*r* = 0.550, *p* ≤ 0.001, Supplemental Figure 1A) and with EMR1 expression (*r* = 0.398, *p* = 0.082, Supplemental Figure 1B).

In line with gene expression results, mincle protein was significantly upregulated in liver samples of NASH patients in comparison to liver samples of control patients (Figure 1E). To further substantiate a close interaction of mincle and fibrosis in NASH, we injected the mincle ligand Trehalose-6,6-dibehenate (TDB) in a murine model of NASH that was established in our lab (18) to stimulate mincle signaling. TDB is a synthetic analog of the mycobacterial cell wall glycolipid TDM. TDB significantly increases hepatic collagen accumulation in comparison to untreated littermates as shown by measurement of the hepatic hydroxyproline, a major component of collagen (Figure 1F).

### Hepatic Mincle in Cirrhosis and ACLF

**Figure 2:**
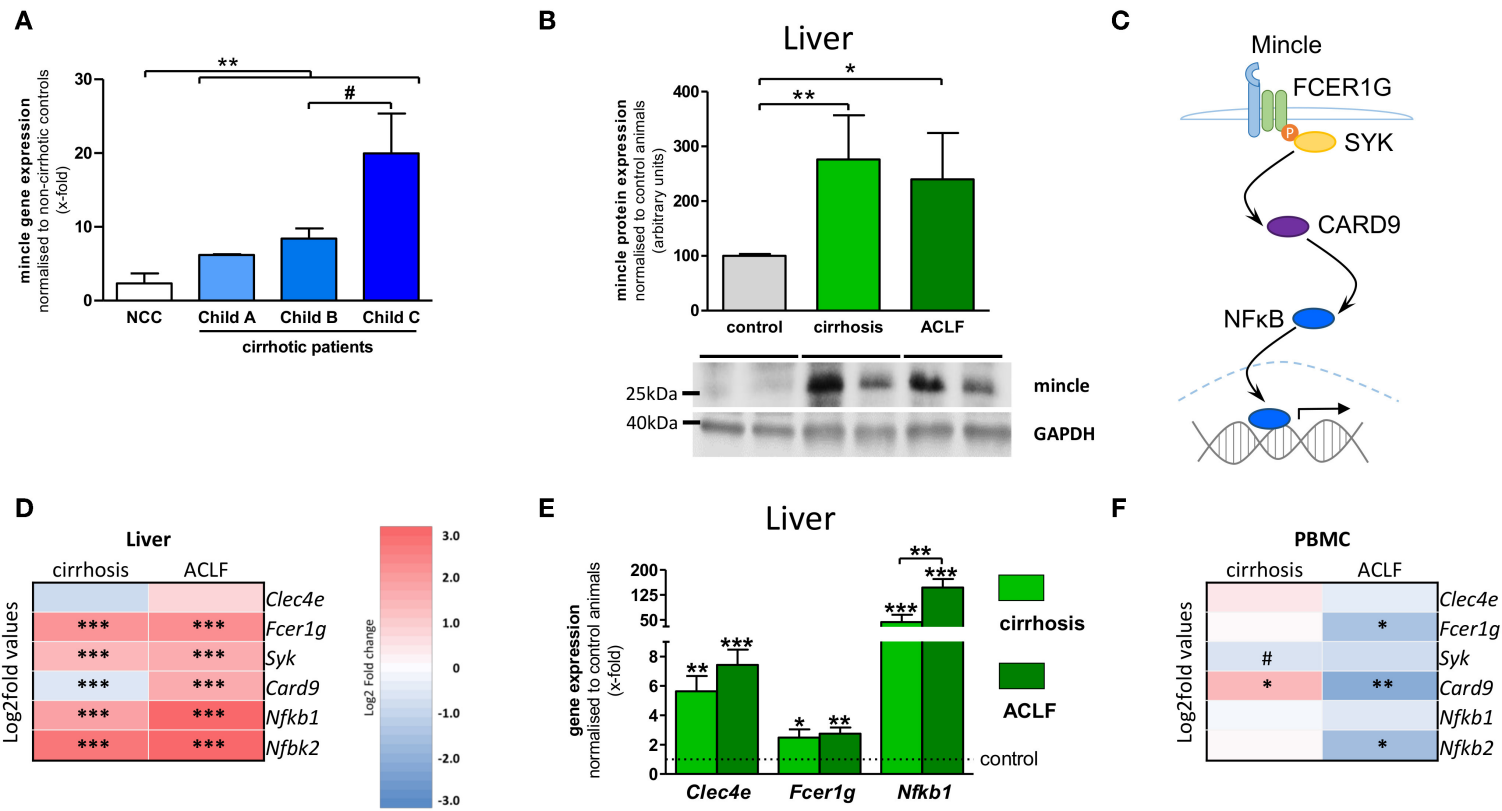
Hepatic mincle in cirrhosis and ACLF. **(A)** Gene expression of mincle (gene *CLEC4E*) in cirrhotic patients stratified by severity of chronic liver disease compared to non-cirrhotic controls (NCC). **(B)** Representative Western blot showing protein levels of mincle in control, cirrhotic induced by bile-duct ligation (BDL), and rats with acute-on-chronic liver failure (ACLF) induced by lipopolysaccharide (LPS) within cirrhosis and quantification of the Western blots. **(C)** Diagram of mincle downstream signaling via FC receptor gamma chain (FCER1G), spleen associated tyrosine kinase (SYK), caspase recruitment domain family member 9 (CARD9) and nuclear factor kappa B (NFκB) leading to expression of pro-inflammatory genes. **(D)** Heatmap of mincle and downstream signaling maker expression in cirrhotic and ACLF livers. **(E)** Gene expression analysis of *Clec4e, Fcer1g*, and *Nfkb1* by qPCR in cirrhotic and ACLF liver compared to sham-operated control. **(F)** Heatmap of mincle and downstream signaling maker expression in cirrhotic and ACLF peripheral blood mononuclear cells (PBMC). Arrows indicate the respective band used for quantification in cases when more than one band is shown in the representative Western blots. In heatmaps, statistical comparison was performed between cirrhosis or ACLF and sham-operated control rats. Upregulation is marked by red colors and downregulation by blue colors. ^*^p < 0.05; ^**^p < 0.01; ^***^p < 0.001; ^#^*p* < 0.1 using non-parametric Mann–Whitney *U t*-test or FDR-adjusted paired *t*-test for data from microarray assay.

Since the upregulation of mincle and its activity enhances hepatic fibrogenesis in NASH, the more severe stages of chronic liver disease were investigated. Mincle expression was highly upregulated in human liver samples of cirrhotic patients compared to donor livers and is associated with the severity of chronic liver disease assessed by Child–Pugh score, reflecting the prognosis of cirrhosis (Figure 2A). Furthermore, hepatic mincle expression in cirrhotic patients is inversely associated with platelet count (*r* = −0.372, *p* = 0.023), as a surrogate for the severity of portal hypertension.

ACLF develops from liver cirrhosis and is associated with systemic inflammation, multi-organ failure, and high short-term mortality. To assess hepatic levels of mincle in this condition, ACLF was triggered by lipopolysaccharide (LPS) administration in a well-established rat model of cholestatic cirrhosis. Protein levels of mincle were increased in liver samples of cirrhotic and ACLF rats in comparison to sham-operated control animals (Figure 2B).

The rodent models of cirrhosis and ACLF were also used to examine downstream signaling of mincle via FC receptor gamma chain (gene *Fcer1g*), spleen associated tyrosine kinase (gene *Syk*), caspase recruitment domain family member 9 (gene *Card9*) and nuclear factor kappa B (genes *Nfkb1* and *NFkb2*) in liver and in circulating immune cells via transcriptome analysis (Figure 2C). In liver samples, the components of the mincle downstream signaling were significantly upregulated in both cirrhosis and ACLF compared to sham-operated control animals (Figure 2D). Upregulation of the downstream signaling in experimental cirrhosis and ACLF were confirmed by qPCR for *Fcer1g* and *Nfkb1* (Figure 2E). In PBMC, the expression profile of mincle signaling was almost unchanged in cirrhosis. By contrast, a clear downregulation of the gene was observed in ACLF (Figure 2F).

There is a clear dysregulation of mincle between liver and PBMCs in cirrhosis and ACLF. Since cirrhosis and ACLF are systemic diseases we also investigated mincle and its downstream signaling in extrahepatic tissues.

### Mincle in Extrahepatic Tissues in Cirrhosis and ACLF

**Figure 3:**
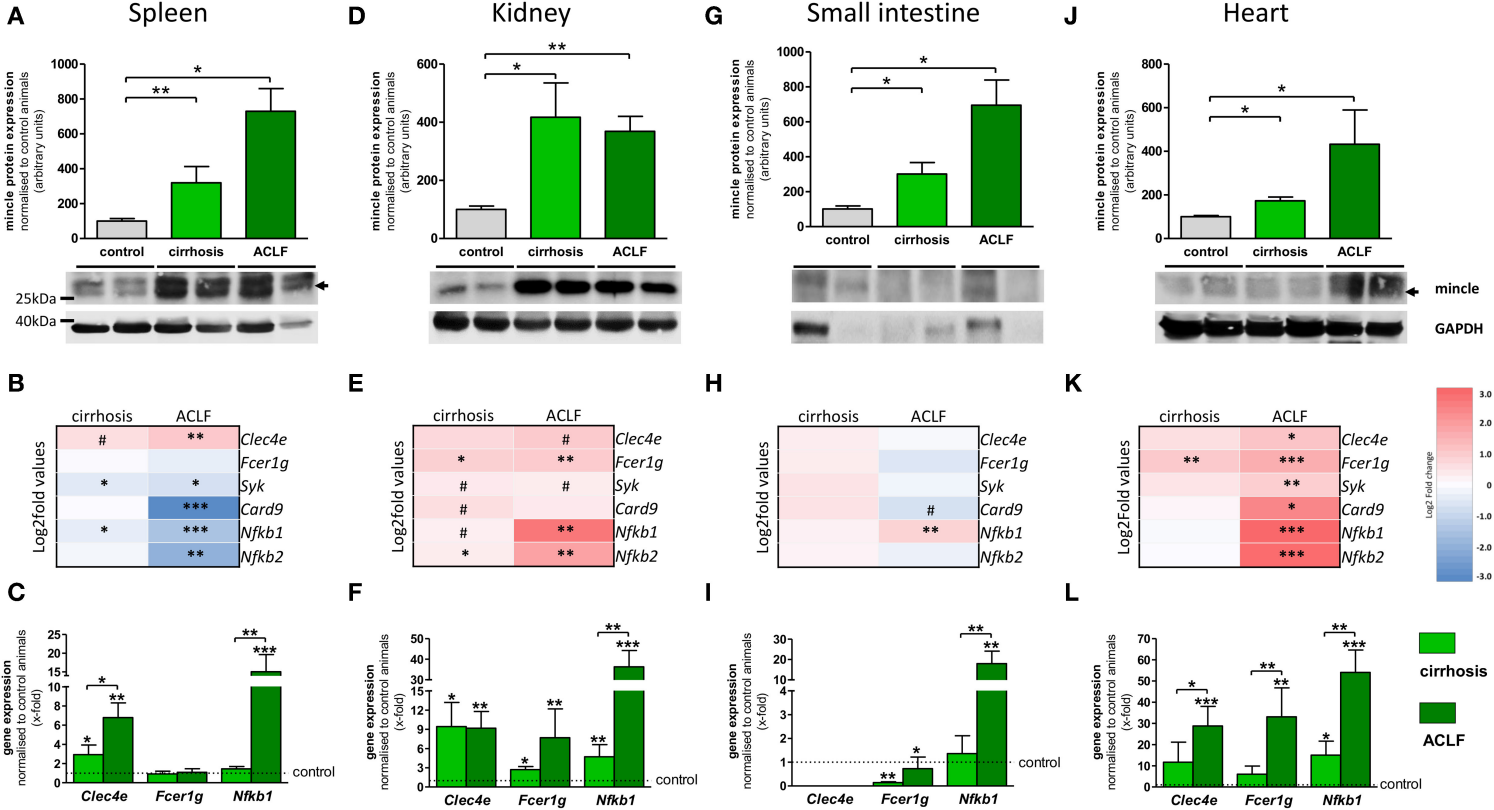
Extrahepatic mincle in cirrhosis and ACLF. **(A–C)** Mincle in the spleen. **(A)** Representative Western blot showing protein levels of mincle in control, cirrhotic, and ACLF rats and quantification of the Western blots. **(B)** Heatmap of mincle and downstream signaling maker expression in cirrhosis and ACLF. **(C)** Gene expression of Clec4e, Fcer1g and Nfkb1 in cirrhosis and ACLF. **(D–F)** Mincle in kidney. **(D)** Representative Western blot showing protein levels of mincle in control, cirrhotic, and ACLF rats and quantification of the Western blots. **(E)** Heatmap of mincle and downstream signaling maker expression in cirrhotic and ACLF. **(F)** Gene expression of Clec4e, Fcer1g, and Nfkb1 in cirrhosis and ACLF. **(G–I)** Mincle in small intestine. **(G)** Representative Western blot showing protein levels of mincle in control, cirrhotic and ACLF rats and quantification of the Western blots. **(H)** Heatmap of mincle and downstream signaling maker expression in cirrhotic and ACLF. **(I)** Gene expression of Clec4e, Fcer1g, and Nfkb1 in cirrhosis and ACLF. **(J–L)** Mincle in heart. **(J)** Representative Western blot showing protein levels of mincle in control, cirrhotic, and ACLF rats and quantification of the Western blots. **(K)** Heatmap of mincle and downstream signaling maker expression in cirrhotic and ACLF. **(L)** Gene expression of Clec4e, Fcer1g, and Nfkb1 in cirrhosis and ACLF. Arrows indicate the respective band used for quantification in cases when more than one band is shown in the representative Western blots. In heatmaps, statistical comparison was performed between cirrhosis, respectively, ACLF and sham-operated control rats. Upregulation is marked by red colors and downregulation by blue colors. ^*^p < 0.05; ^**^p < 0.01; ^***^p < 0.001; ^#^*p* < 0.1 using using non-parametric Mann–Whitney *U t*-test or FDR-adjusted paired *t*-test for data from microarray assay.

Inflammation in cirrhosis and, especially, in ACLF affects extrahepatic organs (kidney, spleen) but is also maintained and triggered by them (e.g., bacterial translocation from the intestine). Therefore, mincle expression and signaling profile were investigated in extrahepatic tissues. In spleen, protein levels of mincle significantly increased in cirrhosis and showed a stronger enhancement in ACLF (Figure 3A). The downstream signaling profile was similar to the one observed in PBMC, with minor changes of gene expression in cirrhosis but a significant downregulation of the signaling components in ACLF (Figure 3B). This could be mainly confirmed in qPCR experiments. However, it seems that there was also a mincle-independent splenic upregulation of *Nfkb1 in ACLF* (Figure 3C). In kidney, protein levels of mincle increased in cirrhosis and in ACLF (Figure 3D). As with higher renal mincle levels in cirrhosis and ACLF, downstream signaling profile showed an upregulation of the components in cirrhosis and in ACLF (Figure 3E) and was confirmed by pPCR (Figure 3F). In tissue samples of the small intestine, protein levels of mincle seemed to bring about a relative increase comparable to the one observed in spleen and kidney tissue samples (Figure 3G). However, in transcriptome analysis of the small intestine, the mincle upregulation failed to translate into an upregulation of the downstream signaling (Figure 3H). Results from qPCR confirmed the transcriptome analysis, except for a mincle-independent upregulation of *Nfkb1* (Figure 3I). In heart tissue, protein levels of mincle showed a similar increase in the disease stages from healthy control via cirrhosis to ACLF (Figure 3J). The downstream signaling profile showed a mild increase, at least in some of the components in cirrhosis, but a marked significant increase in ACLF (Figure 3K). These results could be confirmed by qPCR experiments (Figure 3L).

Taken together, protein levels of mincle are increased in cirrhosis and ACLF in all observed organs, with the highest expression in ACLF. However, this upregulation does not lead to enhanced downstream signaling in all organs. It rather seems that there are two different expression profiles, one that shows a marked upregulation of the mincle downstream signaling, as in liver, kidney, and heart, and one that shows a dysregulation of the downstream signaling, as in PBMC, spleen, and the small intestine. Gene expression experiments revealed a mincle-independent increase of *Nfkb1* in organs with dysregulated downstream signaling, which, however, was not as prominent as *Nfkb1* upregulation in organs with mincle-dependent upregulation of the downstream signaling, such as liver, kidney, and heart.

## Discussion

This study describes for the first time the mincle expression in different stages of chronic liver disease especially in more advanced stages of chronic liver disease and ACLF. Interestingly, the downstream signaling of mincle under these conditions seems not systemic. While mincle signaling is downregulated in compartments of the immune system and the intestinal barrier, it is upregulated in the other observed tissues including the liver.

To date, very little is known about the role of mincle in chronic liver disease, particularly its hepatic function. In NASH, mincle is involved in the formation of crown-like structures, in which infiltrating macrophages attracted from dying adipocytes surround these cells and thereby promote adipose tissue inflammation and fibrosis (10, 11). Similar structures have also been identified in liver tissue in experimental and human NASH when macrophages aggregate around hepatocytes that contain large lipid droplets, which significantly correlated with fibrosis. Of note, these structures were primarily described for NASH—but rarely for viral hepatitis—as a general mechanism for chronic liver diseases. However, the impact of mincle on the progression of liver disease was not investigated (12). This study complements previous findings by providing additional data, especially in human samples and provides evidence that mincle is upregulated in NASH and its pro-fibrotic properties.

Moreover, mincle seems to play a role in chronic liver diseases in general. Our study confirms previous data from the murine model (9) and demonstrates that mincle is also upregulated in more advanced stages of human chronic liver disease and that it is associated with disease progression regardless of etiology. The correlation of mincle expression with Child-Pugh score and platelet count as readouts for portal hypertension, suggest that mincle is associated with the major complication in cirrhosis promoting the development of systemic inflammation and ACLF. Thereby, the degree of activation of systemic inflammation can be used as a predictor for disease progression and mortality. While in decompensated cirrhosis, a partial activation of systemic inflammation with low short-term mortality can be observed, ACLF is characterized by complete activation of systemic inflammation and high short-term mortality (27–29). The upregulation of inflammatory markers is mediated primarily by myeloid cells and is associated with activation of the immune response due to bacterial translocation (30–32). Therefore, the assessment of mincle in ACLF was of special interest for this study, since systemic inflammation concurs with local inflammation in the liver. Interestingly, Martínez-López et al. (15) reported that mincle deficient mice showed increased bacterial translocation into the circulation and increased hepatic inflammation. In fact, the current study demonstrates a dysregulated mincle downstream signaling in compartments of the immune system and intestinal barrier in ACLF, namely PBMC, spleen and the small intestine. In previous studies, we could demonstrate a specific blood microbiome in decompensated cirrhosis (31). Therefore, the dysregulated mincle signaling might contribute to bacterial translocation and the development toward ACLF. Thus, there exists a clear relationship between portal hypertension and development of ACLF mediated by systemic inflammation (13, 33). This relationship is supported by the fact that systemic inflammation can be reduced if the underlying portal hypertension is ameliorated by insertion of a transjugular intrahepatic portosystemic shunt (34–36).

Although advanced chronic liver disease features systemic inflammation, “sepsis-like” immune paralysis develops in more severe stages facilitating secondary infections and organ failure (37, 38). Mincle possibly plays an important role in these apparently opposing observations, since the dysregulation of mincle signaling in spleen and PBMC, as important compartments of the immune system, could be responsible for the failure to clear invading gut bacteria and consequent systemic inflammation. This might also contribute to the compartmentalization of the immune response in decompensated cirrhosis and ACLF (32). This study proves that upregulation of mincle in ACLF is not restricted to the liver but is also found in other important compartments. However, mincle upregulation does not necessarily translate into an upregulation of the downstream signaling via FCER1G/SYK/CARD9/NFκB. Downstream signaling of mincle is differently regulated dependent on the compartment of the immune system and the intestinal barrier. This finding indicates that mincle possibly plays an important role in immune paralysis observed in ACLF. The role of mincle in spleen and small intestine upon progression of chronic liver disease remains uncertain and requires further clarification, since some of the data from the microarray could not be confirmed by qPCR. However, we can not exclude that these discrepancies in the results are due to the two different used methods as lack of convertibility between results generated by microarray or qPCR has been reported (39, 40).

The co-occurrence of complications of advanced chronic liver disease and systemic inflammation leads to extrahepatic organ dysfunction promoting the development of ACLF. Renal dysfunction in cirrhosis leads to ascites formation and implicates inflammation by bacterial infections promoting progression toward ACLF (41). Moreover, kidney failure is the most common additional organ failure in ACLF originating probably from inflammation and systemic hemodynamic instability (42). The combination of vasoconstrictor terlipressin and albumin provides the best treatment, probably by combining hemodynamic as well as immunomodulatory properties (43). Interestingly, in subarachnoid hemorrhage, a subtype of stroke, albumin treatment attenuated activation of the immune response in parenchymal macrophages of the central nervous system (44). Therefore, the interaction of albumin and mincle in tissue-resident macrophages may also be one of the beneficial effects of albumin treatment in patients with advanced chronic liver disease (38).

In addition to renal dysfunction, circulatory dysfunction also plays an important role in the development of ACLF. Reduced heart rate variability is a common feature of cirrhosis due to systemic inflammation. Heart rate variability is further reduced in patients developing ACLF and can be used to predict disease progression and short-term mortality (45). Interestingly, mincle is also expressed in cardiac tissue (46). We also observed an upregulation of mincle in cardiac tissue in the model of ACLF. This may be explained by cell dysfunction, whereby mincle induces necroptosis (46).

The major limitation of this study is that mincle expression was only measured in whole organ lysates but not in the respective resident macrophage populations of each organ.

Mincle could be another integral component in the cross-talk between macrophages and hepatic stellate cells that translates macrophage-induced fibrosis. This study opens avenues for the investigation of the processes behind dysregulated mincle signaling in different compartments of the immune system.

## Data Availability Statement

The raw data supporting the conclusions of this article will be made available by the authors, without undue reservation, to any qualified researcher.

## Ethics Statement

The studies involving human participants were reviewed and approved by ethics committee at the University of Bonn and ethics committees of the North Rhine-Westphalian Chamber of Medicine. The patients/participants provided their written informed consent to participate in this study. The animal study was reviewed and approved by North Rhine-Westphalian State Agency for Nature, Environment, and Consumer Protection (LANUV, Germany).

## Author Contributions

RS, JT, and SK: conceptualization. RS, FU, CO, ST, MB, OT, WG, CG, SZe, AP, PP, SZi, CW, LS, KR, JT, and SK: methodology. RS, FU, CO, ST, JT, and SK: formal analysis. RS, FU, CO, JT, and SK: investigation. RS, JT, and SK: writing-original draft. RS, FU, CO, ST, MB, OT, WG, CG, SZe, AP, PP, SZi, CW, LS, KR, JT, and SK: approved final version of the manuscript. RS, JT, and SK: visualization. RS, JT, and SK: supervision. All authors contributed to the article and approved the submitted version.

## Conflict of Interest

The authors declare that the research was conducted in the absence of any commercial or financial relationships that could be construed as a potential conflict of interest.
