# Supplementary material for: The Role of Macrophage-Inducible C-Type Lectin in Different Stages of Chronic Liver Disease
Source: Front Immunol. 2020 Jul 7;11:1352. doi: 10.3389/fimmu.2020.01352 (PMC7358277; doi:10.3389/fimmu.2020.01352)
Supplement: Supplementary file 1 [file Data_Sheet_1.docx]

**The role of macrophage-inducible C-type lectin in different stages of chronic liver disease**

Robert Schierwagen ^1^, Frank E. Uschner ^1^, Cristina Ortiz ^1^, Sandra Torres ^1^, Max J. Brol ^2^, Olaf Tyc ^1^, Wenyi Gu ^1^, Christian Grimm ^1^, Stefan Zeuzem ^1^, Andreas Plamper ^3^, Philipp Pfeifer ^4^, Sebastian Zimmer ^4^, Christoph Welsch ^1^, Liliana Schaefer ^5^, Karl P. Rheinwalt ^3^, Joan Clària ^6^, Vicente Arroyo ^6^, Jonel Trebicka ^1,6,7,8^, Sabine Klein ^1^

**Affiliations:**

^1^ Department of Internal Medicine I, University Hospital, Goethe University, Frankfurt, Germany

^2^ Department of Internal Medicine I, University of Bonn, Bonn, Germany

^3^ Department for Bariatric, Metabolic and Plastic Surgery, St. Franziskus-Hospital, Cologne, Germany

^4^ Department of Medicine II, Heart Center, University Hospital Bonn, Bonn, Germany

^5^ Centre for Pharmacy Frankfurt/ZAFES, Institute for Pharmacology and Toxicology, University Hospital, Goethe University, Frankfurt, Germany

^6^ European Foundation for the Study of Chronic Liver Failure, Barcelona, Spain

^7^ Department of Medical Gastroenterology and Hepatology, Odense University Hospital, Odense, Denmark

^8^ Institute for Bioengineering of Catalonia, Barcelona, Spain

**Correspondence:**

Jonel Trebicka Department of Internal Medicine I, University Hospital, Goethe University, Frankfurt, Germany, Theodor-Stern-Kai 7, 60590 Frankfurt. jonel.trebicka@kgu.de, Tel: + 49 69 6301 4256

**Keywords:** ACLF, bacterial translocation, fibrosis, inflammation, NASH

**Running Title:** Mincle in chronic liver disease

**Supplemental data**

**
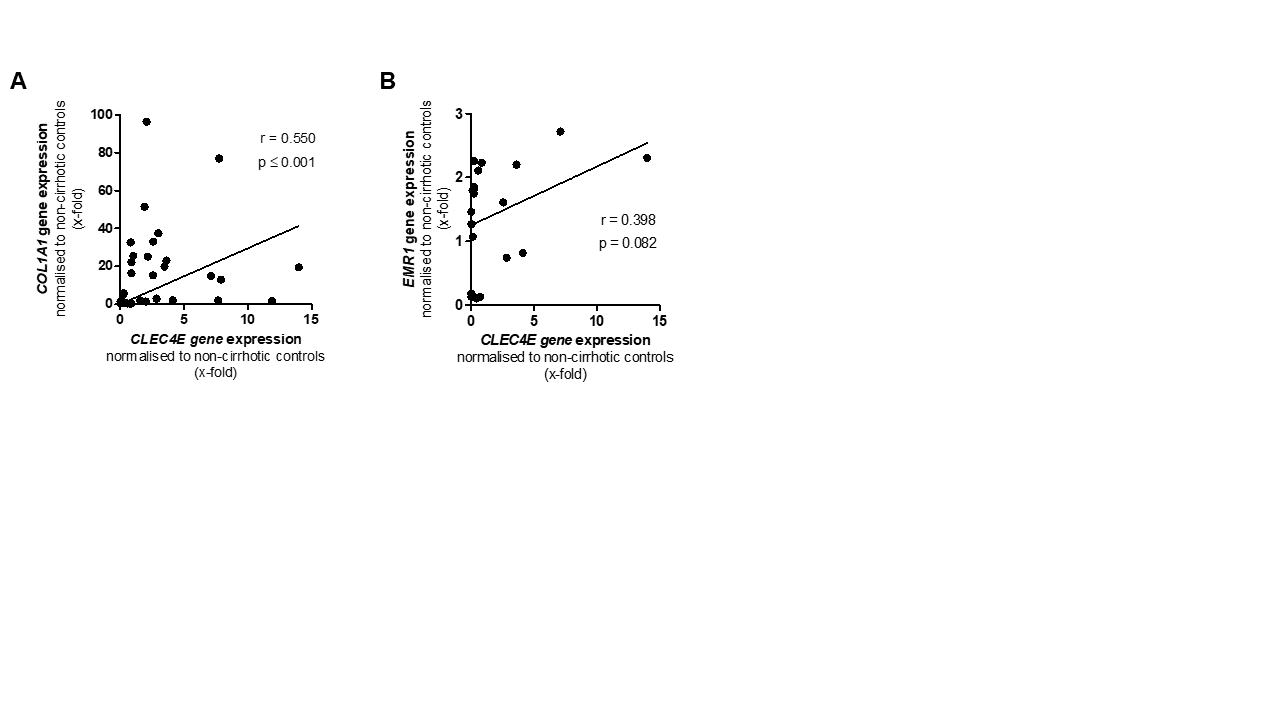
**

**Supplemental Figure 1:** (A) Correlation matrix of *COL1A1* and *CLEC4E* expression in humans with and without non-alcoholic steatohepatitis (NASH). (B) Correlation matrix of *EMR1* and *CLEC4E* expression in humans with and without non-alcoholic steatohepatitis (NASH).

**Supplemental Table 1: Taqman assays**

| Gene | Assay ID | Species |
| --- | --- | --- |
| *ACTA2* | Hs00426835_g1 | Human |
| *ADGRE1* | Hs00892591_m1 | Human |
| *CLEC4E* | Hs00372017_m1 | Human |
| *Clec4e* | Rn01492266_m1 | *Rattus norvegicus* |
| *COL1A1* | Hs00164004_m1 | Human |
| *Fcer1g* | Rn01411789_m1 | *Rattus norvegicus* |
| *Nfkb1* | Rn01399572_m1 | *Rattus norvegicus* |
